# Simulation-based training using a novel Surabaya hysterectomy mannequin following video demonstration to improve abdominal hysterectomy skills of obstetrics and gynecology residents during the COVID-19 pandemic in Indonesia: a pre- and post-intervention study

**DOI:** 10.3352/jeehp.2022.19.11

**Published:** 2022-05-17

**Authors:** Dara Dasawulansari Syamsuri, Brahmana Askandar Tjokroprawiro, Eighty Mardiyan Kurniawati, Budi Utomo, Djoko Kuswanto

**Affiliations:** 1 Department of Obstetrics and Gynecology, Dr. Soetomo General Academic Hospital, Medical Faculty, Universitas Airlangga, Surabaya, Indonesia; 2 Department of Public Health-Preventive Medicine, Medical Faculty, Universitas Airlangga, Surabaya, Indonesia; 3 Integrated Digital Design Laboratory, Industrial Design Department, Institut Teknologi Sepuluh Nopember, Surabaya, Indonesia; Hallym University, Korea

**Keywords:** Gynecology, Hysterectomy, Indonesia, Manikins, Simulation training

## Abstract

**Purpose:**

During the coronavirus disease 2019 (COVID-19) pandemic, the number of abdominal hysterectomy procedures decreased in Indonesia. The existing commercial abdominal hysterectomy simulation model is expensive and difficult to reuse. This study compared residents’ abdominal hysterectomy skills after simulation-based training using the Surabaya hysterectomy mannequin following a video demonstration.

**Methods:**

We randomized 3rd- and 4th-year obstetrics and gynecology residents to a video-based group (group 1), a simulation-based group (group 2), and a combination group (group 3). Abdominal hysterectomy skills were compared between before and after the educational intervention. The pre- and post-tests were scored by blinded experts using the validated Objective Structured Assessment of Technical Skills (OSATS) and Global Rating Scale (GRS).

**Results:**

A total of 33 residents were included in the pre- and post-tests. The OSATS and GRS mean differences after the intervention were higher in group 3 than in groups 1 and 2 (OSATS: 4.64 [95% confidence interval [CI], 2.90–6.37] vs. 2.55 [95% CI, 2.19–2.90] vs. 3.82 [95% CI, 2.41–5.22], P=0.047; GRS: 10.00 [95% CI, 7.01–12.99] vs. 5.18 [95% CI, 3.99–6.38] vs. 7.18 [95% CI, 6.11–8.26], P=0.006). The 3rd-year residents in group 3 had greater mean differences in OSATS and GRS scores than the 4th-year residents (OSATS: 5.67 [95% CI, 2.88–8.46]; GRS: 12.83 [95% CI, 8.61–17.05] vs. OSATS: 3.40 [95% CI, 0.83–5.97]; GRS: 5.67 [95% CI, 2.80–8.54]).

**Conclusion:**

Simulation-based training using the Surabaya hysterectomy mannequin following video demonstration can be a bridge to learning about abdominal hysterectomy for residents who had less surgical experience during the COVID-19 pandemic.

## Introduction

### Background

**Fig. 1.**
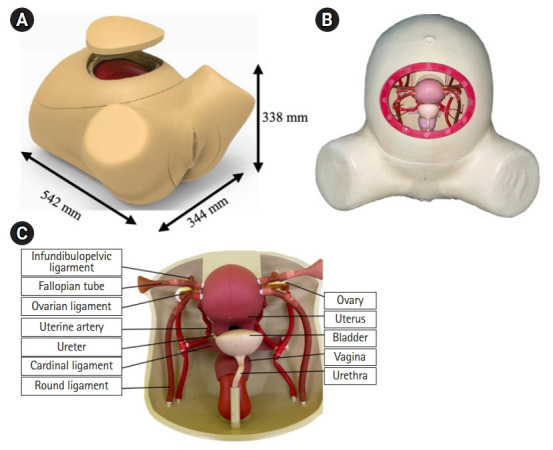
Surabaya hysterectomy mannequin. (A) Main body model. (B) Internal genitalia simulation model in the main body. (C) Uterine assembly.

The coronavirus disease 2019 (COVID-19) pandemic has made it difficult for residents to train with large numbers of patients, particularly in obstetrics and gynecology. The majority of respondents in Nigeria said they saw fewer patients in outpatient clinics (83.6%), as well as fewer emergency and elective surgical procedures (58.5% and 90.8%, respectively) in 2020 [1]. According to data from Indonesia Soetomo Hospital’s operating room registration, obstetric hysterectomy operations decreased by 38.3% in 2021 compared to 2019 (before the COVID-19 pandemic), while gynecological hysterectomy operations decreased by 57.6% in 2020 and 51.7% in 2021 compared to 2019 [2]. Adapting to the COVID-19 era to the 3 pillars of surgical resident education, video-based and recorded teaching sessions can be used to replace traditional in-person lectures and didactic sessions, the use of home-based skills recording and remote evaluation, simulation, and skills development complies with appropriate social distancing guidelines, and peer-reviewed video-based education modules can be used for operative education and preparation [3]. The existing commercial abdominal hysterectomy simulation model for obstetrics and gynecology residents is expensive and difficult to reuse. The not-for-profit Surabaya hysterectomy mannequin that we made and employed in this study is an internal genitalia simulation model that may be used to simulate abdominal hysterectomy surgery (Fig. 1). This simulation model can be used to conduct steps such as incisions in the abdominal lining, cutting the uterine ligament, removing the uterus, and closing the abdominal wall. This Surabaya hysterectomy mannequin can be used several times. The design is meant to correspond to women of average size and can be readily moved.

### Objectives

This study aimed to compare residents’ abdominal hysterectomy skills using the validated Objective Structured Assessment of Technical Skills (OSATS) and Global Rating Scale (GRS) among obstetrics and gynecology residents, before and after a planned educational program. Furthermore, it compared the effectiveness of 3 different intervention strategies: video demonstration, simulation-based training using the Surabaya hysterectomy mannequin, and both interventions.

## Methods

### Ethics statement

The research ethics committee of Universitas Airlangga’s affiliated hospital approved this study (approval no., 0271/KEPK/X/2021). Before taking part in this study, participants gave their informed consent.

### Study design

**Fig. 2.**
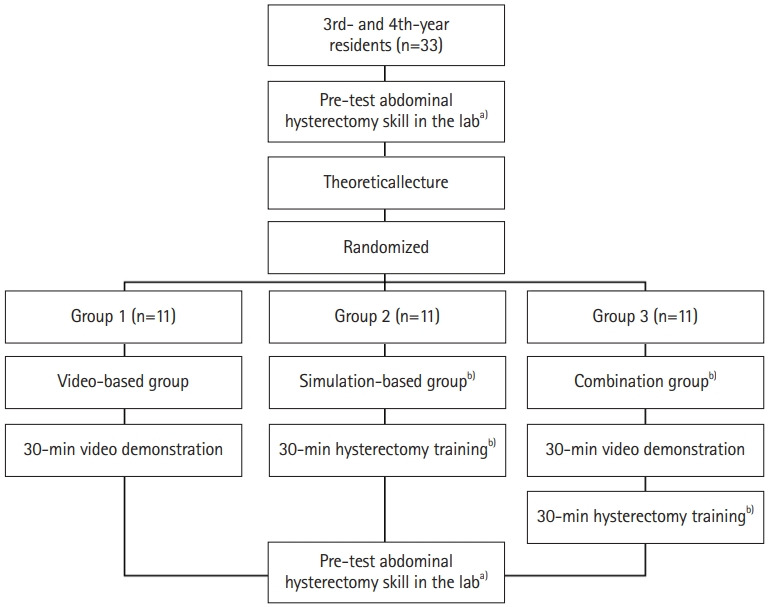
Study design. ^a)^Scored using validated assessment tools: the Objective Structured Assessment of Technical Skills and the Global Rating Scale. ^b)^Using the Surabaya hysterectomy mannequin.

This study used a quasi-experimental design (pre- and post-test studies). The flow diagram is presented in Fig. 2. It was described according to the STROBE (Strengthening the Reporting of Observational Studies in Epidemiology) guideline, available from: https://www.strobe-statement.org.

### Setting

Universitas Airlangga’s 3rd- and 4th-year obstetrics and gynecology residents were approached for participation in the study between October and December 2021 at the Department of Obstetrics and Gynecology, Dr. Soetomo General Academic Hospital in Surabaya, Indonesia. For group assignment, we used a computer-generated randomization list, with stratified proportionate random sampling. Thirty-three residents were randomly assigned to 1 of 3 groups based on their residency year level (3rd and 4th year) until all 3 groups had an equal number of subjects. All participants were rated on their performance of abdominal hysterectomy as a baseline technical skill (pre-test). After that, all participants received a lecture about abdominal hysterectomy. Participants in each group received a different educational intervention. The first group (the video-based group) was given a video demonstration (Supplement 1), the second group (the simulation-based group) was given hysterectomy training using a Surabaya hysterectomy mannequin, and the third group (the combination group) received both interventions sequentially. One week after the intervention under expert supervision, a technical skill evaluation was conducted (post-test). In summary, blinded experts graded the pre- and post-test in a laboratory setting utilizing the Surabaya hysterectomy mannequin using the validated OSATS and GRS. The expert reviewer was unaware of whether the participant they rated were pre- or post-intervention, as well as their randomization status.

### Participants

Thirty-three obstetrics and gynecology residents in their 3rd and 4th years were invited (Table 1). The study included residents with no prior experience of performing abdominal hysterectomy on patients independently. There were no exclusion criteria.

### Variables

Before and after the educational intervention, 2 variables were examined in this study: OSATS and GRS. The OSATS is an objective structured approach of evaluation that is used to evaluate a variety of specific surgical techniques. The form is intended to allow the assessor to interpret the entire procedure, allowing for an objective assessment [1].

### Data sources/measurement

To assess abdominal hysterectomy procedure-specific skills, we developed the OSATS form for total abdominal hysterectomy (TAH) (Supplement 2) with 11 procedural steps: (1) performing laparotomy and developing the visual field; (2) ligating and cutting the round ligament; (3) incising the anterior leaf of the broad ligament; (4) clamping, cutting, and ligating the ovarian ligament and fallopian tube (or the infundibulopelvic ligament); (5) mobilizing the bladder; (6) clamping, cutting, and ligating the uterine artery and vein; (7) clamping, cutting, and ligating the cardinal ligament/sacrouterine ligament; (8) removing the uterus; (9) closing the vaginal cuff; (10) performing hemostasis; and (11) closing the abdominal wall. The OSATS checklist score is based on a 2-point scale (0=needs help, 1=performed independently) for the aforementioned criteria, with a maximum of 11. We created and conducted this OSATS validation study (Tables 2, 3).

The GRS was the second variable examined in this study (Supplement 3). This is a form on which the assessor must decide if the trainee has shown ability and competence in each of the 7 generic technical skills assessment domains that are common to all OSATS procedures [4]. Performance is graded on a 1 to 5 Likert scale (1=very poor, 2=poor, 3=average, 4=good, 5=excellent) in each category, with a total possible score of 35. The 7 generic technical skills assessment domains are (1) respect for tissue, (2) time and motion, (3) instrument handling, (4) knowledge of the instrument, (5) flow of the operation, (6) use of assistants, and (7) knowledge of the specific procedure. We created and conducted this GRS validation study (Tables 4, 5).

### Intervention

#### Video demonstration

For the video demonstration, we used the same hysterectomy mannequin that was used in the study (Surabaya hysterectomy mannequin). Participants allocated to group 1 or group 3 received a 30-minute video demonstration session, watching an instructional video demonstrating TAH procedures. The video was made by our department and utilized for resident training purposes. One of the authors demonstrated all 11 steps of performing a TAH according to the OSATS checklist used in this study.

#### Simulation-based training

According to the OSATS checklist used in this study, participants in groups 2 and 3 (group 3 after the video demonstration session) executed all 11 steps of TAH. The participants performed their abdominal hysterectomy on a Surabaya hysterectomy mannequin under expert supervision.

#### Simulator development of the Surabaya hysterectomy mannequin

In the Integrated Digital Design Laboratory, Industrial Design Department, Institut Teknologi Sepuluh Nopember, from April to September 2021, we created a simulation model of abdominal hysterectomy using Fusion360TM (Autodesk Inc., San Rafael, CA, USA) that was adjusted to normal human size with reference to anatomy books. We utilized a 3-dimensional (3D) printer to create a mold, then filled with room-temperature-vulcanizing silicone rubber for the uterus, ovarium, cervix, vagina, and abdominal wall, and latex for the ligaments and arteries. This simulation model can be replaced every time we perform an abdominal hysterectomy (Fig. 1). This simulation model is a product that has not been commercialized.

### Bias

There was a low risk of selection and performance bias. For group assignment, we used stratified proportionate random sampling. We collected data using a validated instrument and the blinded experts was unaware of whether the participant they rated were pre- or post-intervention, as well as their randomization status

### Study size

There was no estimation of the sample size because all obstetrics and gynecology residents who matched the inclusion criteria and agreed to participate in this study were included. In this study, 33 residents completed both a pre- and post-intervention encounter in which they executed the skill.

### Statistical methods

Data were analyzed using IBM SPSS ver. 26.0 (IBM Corp., Armonk, NY, USA). The Kruskal-Wallis test was used to determine the significance of differences between the 3 groups to evaluate whether residents’ skills improved. P-values <0.05 were considered to indicate statistical significance.

## Results

### Participants and descriptive data

Pre- and post-test data were collected from 33 residents. The mean age was 31.64±2.541 years in group 1, 30.90±2.119 years in group 2, and 30.82±2.960 years in group 3 (Table 1). The distribution of participants according to residency year level was nearly equal (3rd-year residents in group 1=5, group 2=5, and group 3=6; 4th-year residents in group 1=6, group 2=6, and group 3=5). Four residents had never assisted in a TAH, 12 residents had assisted in a TAH 1 to 5 times, 14 residents had assisted in a TAH 6 to 10 times, and 3 residents had assisted in a TAH more than 10 times. Nine residents had performed TAH 1 to 5 times under supervision, while 24 residents had never performed TAH. There was no significant difference (P>0.005) in age, gender, residency year, frequency of having assisted in a TAH, and frequency of having performed TAH under supervision. The raw data file is available in Dataset 1.

### Main results

Before and after the intervention, all participants were graded on their ability to execute an abdominal hysterectomy procedure. Group 1 had a mean OSATS pre-test score of 6.64±2.335, 5.91±2.700 for group 2, and 5.82±3.027 for group 3 (maximum score=11), with no significant difference (P=0.718) (Table 6). The post-test mean increased in all 3 groups, with the highest score in group 3 (group 1: 9.18±2.359, group 2: 9.73±1.849, and group 3: 10.45±0.688) and no significant difference (P=0.487). A significant difference between the pre- and post-test scores was found (P=0.047) (Table 7). Group 3 had the difference in scores (group 1: 2.55 [95% confidence interval (CI), 2.19–2.90]; group 2: 3.82 [95% CI, 2.41–5.22]; and group 3: 4.64 [95% CI, 2.90–6.37]). When compared to 4th-year residents, 3rd-year residents had lower OSATS scores (group 1: 7.60±2.702, group 2: 9.00±2.550, and group 3: 10.17±0.753), but higher differences in scores (group 2: 5.20 [95% CI, 2.37–8.03] and group 3: 5.67 [95% CI, 2.88–8.46]). The raw data file is available in Dataset 2.

Group 1 had a mean GRS pre-test score of 23.36±4.342, 21.27±4.496 for group 2, and 22.27±5.159 for group 3 (maximum score=35), with no significant difference (P=0.567) (Table 8). The post-test mean increased in all 3 groups, with group 3 having the highest score (group 1: 28.55±4.367, group 2: 28.45±3.934, and group 3: 32.27±1.272), with a significant difference (P=0.014). A significant difference (P=0.006) was discovered when the difference between the pre- and post-test scores was compared (Table 9). The largest difference in the highest scores was seen in group 3 (group 1: 5.18 [95% CI, 3.99–6.38]; group 2: 7.18 [95% CI, 6.11–8.26]; and group 3: 10.00 [95% CI, 7.01–12.99]). The 3rd-year residents showed lower GRS scores (group 1: 26.00±4.690; group 2: 26.00±4.743; and group 3: 32.00±0.894), but higher differences in scores (group 1: 5.60 [95% CI, 4.49–6.71]; group 2: 7.80 [95% CI, 6.44–9.16]; and group 3: 12.83 [95% CI, 8.61–17.05]) than the 4th-year residents. The raw data file is available in Dataset 3. Summary data for the data analysis, including year level, group, pre- and post-scores for the OSATS, and pre- and post-scores for the GRS was presented in Dataset 4.

**Fig. 3.**
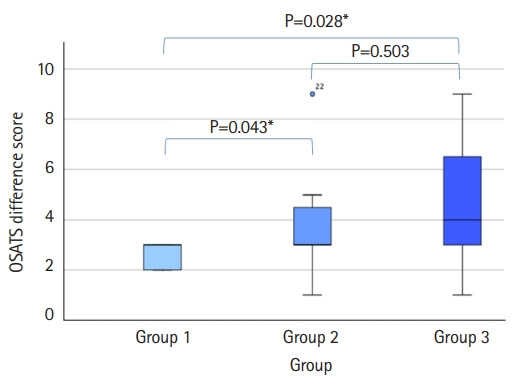
Mean differences in Objective Structured Assessment of Technical Skills scores, with P-values for pairwise comparisons. *P<0.05.

**Fig. 4.**
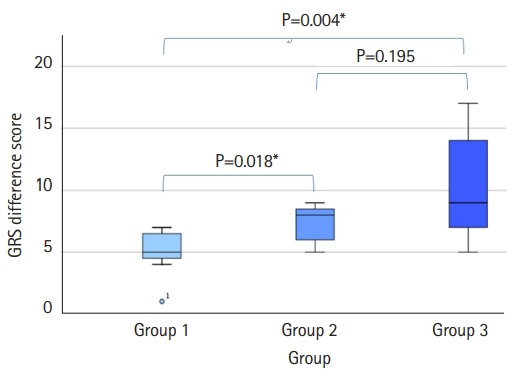
Mean differences in Global Rating Scale scores, with P-values for pairwise comparisons. *P<0.05.

We discovered significant differences between groups 1 and 2 (OSATS, P=0.043; GRS, P=0.018) and groups 1 and 3 (OSATS, P=0.028; GRS, P=0.004) when we compared the pre- and post-test differences between the 2 groups (Figs. 3, 4).

## Discussion

### Key results

When compared to learning with a video demonstration, simulation-based training using the Surabaya hysterectomy mannequin enhanced skill levels to a greater extent. When paired with a video demonstration, simulation-based training using the Surabaya hysterectomy mannequin will improve obstetrics and gynecology residents’ understanding of abdominal hysterectomy.

### Interpretation

This is the first observational study in Indonesia employing a 3D printed mold to create an abdominal hysterectomy simulation model. We found that simulation-based training using the Surabaya hysterectomy mannequin enhanced skill levels to a greater extent than learning with a video demonstration. The 3rd-year residents, who had the least experience with the hysterectomy procedure, showed the greatest impact of simulation-based training. This presents an important lesson in the context of the COVID-19 pandemic, where the number of patients and surgical procedures has decreased. Unlike prior research that employed craft-store supplies to create simulators, the Surabaya hysterectomy mannequin was built of silicone rubber and latex, and the clamping, cutting, ligating, knot-tying, and suturing procedures could all be conducted as they would be in a real situation.

### Comparison with previous studies

Stickrath and Alston [5] presented a study in 2015 that found that using craft store items to make a low-fidelity abdominal hysterectomy simulator increased residents’ confidence in the procedure. The simulator improved residents’ confidence in abdominal hysterectomy, especially among junior residents [5]. Research conducted by Hilal et al. [6] in 2017 showed that residents who completed training using a simulation model experienced improvements in performance compared to residents who received a video demonstrations. Hands-on training improves residents’ technical skills [6]. Surgeons may be able to unleash the benefits of video-based peer coaching and expand the scope of surgical quality improvement [7]. Modern surgical simulators can be used to improve manual dexterity, instill strategies for resolving difficult situations through repeated hysterectomies in a non-life-threatening environment, improve the operative survey, including the ability to make quick and correct decisions, and find strategies for preventing and managing iatrogenic complications [8].

### Limitations

We were unable to compare the study’s findings to real-world residents’ skills and outcomes.

### Generalizability

The results of this study may be useful for other obstetrics and gynecology residents in Indonesia.

### Suggestions

More study is needed to evaluate obstetrics and gynecology residents’ ability to perform an abdominal hysterectomy on real patients.

### Conclusion

Simulation-based training using the Surabaya hysterectomy mannequin following a video demonstration can be a bridge to learning about abdominal hysterectomy for residents who had less surgical experience during the COVID-19 pandemic.

## Figures and Tables

**Table 1.** Participant characteristics in the video-based group (group 1), simulation-based group (group 2), and combination group (group 3)

Characteristic	Group 1	Group 2	Group 3	P-value
Age (yr)	31.64±2.541	30.90±2.119	30.82±2.960	0.717
Gender				0.423
Male	7 (63.6)	8 (72.7)	5 (45.5)	
Female	4 (36.4)	3 (27.3)	6 (54.5)	
Residency year				0.889
3rd year	5 (45.5)	5 (45.5)	6 (54.5)	
4th year	6 (54.5)	6 (54.5)	5 (45.5)	
Frequency of assistance in TAH				0.922
None	1 (9.1)	2 (18.2)	1 (9.1)	
1-5	4 (36.4)	3 (27.3)	5 (45.5)	
6-10	5 (45.5)	6 (54.5)	3 (27.3)	
> 10	1 (9.1)	0	2 (18.2)	
Frequency of TAH performed under supervision				1.000
None	8 (72.7)	8 (72.7)	8 (72.7)	
1-5	3 (33.3)	3 (33.3)	3 (33.3)	
6-10	0	0	0	
>10	0	0	0	

Values are presented as mean±standard deviation or number (%). TAH, total abdominal hysterectomy.

**Table 2.** Validity test of the Objective Structured Assessment of Technical Skills

No.	Checklist	Pearson correlation value	Interpretation
1	Laparotomy and development of the visual field	0.6713	Valid
2	Ligate and cut the round ligament	0.7018	Valid
3	Incises the anterior leaf of the broad ligament	0.6850	Valid
4	Clamp, cut, and ligate the ovarian ligament and fallopian tube (or the infundibulopelvic ligament)	0.6713	Valid
5	Mobilize the bladder	0.7018	Valid
6	Clamp, cut, and ligate the uterine artery and vein	0.8222	Valid
7	Clamp, cut, and ligate the cardinal ligament/sacrouterine ligament	0.8035	Valid
8	Remove the uterus	0.9307	Valid
9	Close the vaginal cuff	0.8222	Valid
10	Perform hemostasis	0.8035	Valid
11	Close the abdominal wall	0.8222	Valid

**Table 3.** Reliability test of the Objective Structured Assessment of Technical Skills

No. of items	Cronbach’s α	Correlation coefficient	Interpretation
11	0.719	0.6319	Reliable

**Table 4.** Validity test of the Global Rating Scale

No.	Checklist	Pearson correlation value	Interpretation
1	Respect for tissue	0.8131	Valid
2	Time and motion	0.7497	Valid
3	Instrument handling	0.7696	Valid
4	Knowledge of instrument	0.7635	Valid
5	Flow of operation	0.7583	Valid
6	Use of assistants	0.8589	Valid
7	Knowledge of specific procedure	0.7497	Valid

**Table 5.** Reliability test of the Global Rating Scale

No. of items	Cronbach’s α	Correlation coefficient	Interpretation
7	0.884	0.6319	Reliable

**Table 6.** Pre- and post-test scores for the Objective Structured Assessment of Technical Skills in the video-based group (group 1), simulation-based group (group 2), and combination group (group 3)

	Group 1		Group 2		Group 3		P-value	
	Pre-test	Post-test	Pre-test	Post-test	Pre-test	Post-test	Pre-test	Post-test
Mean	6.64±2.335	9.18±2.359	5.91±2.700	9.73±1.849	5.82±3.027	10.45±0.688	0.718	0.487
3rd year	5.20±2.864	7.60±2.702	3.80±2.168	9.00±2.550	4.50±3.209	10.17±0.753	0.702	0.244
4th year	7.83±0.753	10.50±0.837	7.67±1.633	10.33±0.816	7.40±2.074	0.80±0.447	0.904	0.569

Values are presented as mean±standard deviation.

**Table 7.** Pre- and post-test differences in the Objective Structured Assessment of Technical Skills scores

	Group 1	Group 2	Group 3	P-value
Mean difference	2.55 (2.19–2.90)	3.82 (2.41–5.22)	4.64 (2.90–6.37)	**0.047**
3rd year	2.40 (1.72–3.08)	5.20 (2.37–8.03)	5.67 (2.88–8.46)	**0.018**
4th year	2.67 (2.12–3.21)	2.67 (1.58–3.75)	3.40 (0.83–5.97)	0.877

Values are presented as mean (95% confidence interval). The bold type is considered statistically significant.

**Table 8.** Mean pre- and post-test scores of the Global Rating Scale in the video-based group (group 1), simulation-based group (group 2), and combination group (group 3)

	Group 1		Group 2		Group 3		P-value	
	Pre-test	Post-test	Pre-test	Post-test	Pre-test	Post-test	Pre-test	Post-test
Mean	23.36±4.342	28.55±4.367	21.27±4.496	28.45±3.934	22.27±5.159	32.27±1.272	0.567	**0.014**
3rd year	20.40±4.930	26.00±4.690	18.20±4.087	26.00±4.743	19.17±4.446	32.00±0.894	0.816	0.055
4th year	25.75±0.957	31.00±3.367	26.00±1.000	31.33±0.577	27.33±2.082	33.00±1.000	0.365	0.139

Values are presented as mean±standard deviation or number (%). The bold type is considered statistically significant.

**Table 9.** Pre- and post-test differences in the Global Rating Scale scores

	Group 1	Group 2	Group 3	P-value
Mean difference	5.18 (3.99–6.38)	7.18 (6.11–8.26)	10.00 (7.01–12.99)	**0.006**
3rd year	5.60 (4.49–6.71)	7.80 (6.44–9.16)	12.83 (8.61–17.05)	**0.005**
4th year	5.25 (0.68–9.82)	5.33 (3.90–6.77)	5.67 (2.80–8.54)	0.357

Values are presented as mean (95% confidence interval). The bold type is considered statistically significant.

## Footnotes

**Data availability**
Data files are available from Harvard Dataverse: https://doi.org/10.7910/DVN/QVMTNM Dataset 1. Raw data of participant characteristic.

Dataset 2. Raw data of participant Objective Structured Assessment of Technical Skills (OSATS) scores before and after the educational intervention.

Dataset 3. Raw data of participant Global Rating Scale (GRS) scores before and after the educational intervention.

Dataset 4. Summary data for the data analysis, including year level, group, pre- and post-scores for the Objective Structured Assessment of Technical Skills (OSATS), and pre- and post-scores for the Global Rating Scale (GRS).

## Supplementary materials

Data files are available from Harvard Dataverse: https://doi.org/10.7910/DVN/QVMTNM.

Supplement 1. Video demonstration of total abdominal hysterectomy using Surabaya hysterectomy mannequin by Department of Obstetrics and Gynecology, Medical Faculty, Universitas Airlangga, Soetomo Hospital, Surabaya, Indonesia.

Supplement 2. Objective Structured Assessment of Technical Skills form for total abdominal hysterectomy (TAH-OSATS).

Supplement 3. Global Rating Scale of operative performance form.

Supplement 4. Audio recording of the abstract.
