# Supplementary material for: Simulation-based training using a novel Surabaya hysterectomy mannequin following video demonstration to improve abdominal hysterectomy skills of obstetrics and gynecology residents during the COVID-19 pandemic in Indonesia: a pre- and post-intervention study
Source: J Educ Eval Health Prof. 2022 May 17;19:11. doi: 10.3352/jeehp.2022.19.11 (PMC9149772; doi:10.3352/jeehp.2022.19.11)
Supplement: Supplementary file 6 — Supplement 2. Objective Structured Assessment of Technical Skills form for total abdominal hysterectomy (TAH-OSATS). [file jeehp-19-11-suppl2.docx]

Objective Structured Assessment of Technical Skills Form for Total Abdominal Hysterectomy (TAH-OSATS)

| Trainee Name: |  | Residency year level: |  |
| --- | --- | --- | --- |
| Assessor Name: |  | Date: |  |
| Clinical details of complexity/difficulty of case  Instrument used: |  | | |

|  | Performed independently | Needs help |
| --- | --- | --- |
|  | PLEASE TICK RELEVANT BOX | |
| Laparotomy and development of the visual field |  |  |
| Ligate and cut the round ligament |  |  |
| Incises the anterior leaf of the broad ligament |  |  |
| Clamp, cut, and ligate the ovarian ligament and fallopian tube (or the infundibulopelvic  ligament) |  |  |
| Mobilize the bladder |  |  |
| Clamp, cut, and ligate the uterine artery and vein |  |  |
| Clamp, cut, and ligate the cardinal ligament/sacrouterine ligament |  |  |
| Remove the uterus |  |  |
| Close the vaginal cuff |  |  |
| Perform hemostasis |  |  |
| Close the abdominal wall |  |  |
| Comments: |  |  |
