# Supplementary material for: Simulation-based training using a novel Surabaya hysterectomy mannequin following video demonstration to improve abdominal hysterectomy skills of obstetrics and gynecology residents during the COVID-19 pandemic in Indonesia: a pre- and post-intervention study
Source: J Educ Eval Health Prof. 2022 May 17;19:11. doi: 10.3352/jeehp.2022.19.11 (PMC9149772; doi:10.3352/jeehp.2022.19.11)
Supplement: Supplementary file 7 — Supplement 3. Global Rating Scale of operative performance form. [file jeehp-19-11-suppl3.docx]

# **GLOBAL RATING SCALE OF OPERATIVE PERFORMANCE**

# Please circle the number corresponding to the candidate's performance in each category, irrespective of training level.

| **Respect for Tissue:** | | | | |
| --- | --- | --- | --- | --- |
| **1** | **2** | **3** | **4** | **5** |
| Frequently used unnecessary force on tissue or caused damage by inappropriate use of instruments |  | Careful handling of tissue but occasionally caused inadvertent damage |  | Consistently handled tissues appropriately with minimal damage |
| **Time and Motion:** | | | | |
| **1** | **2** | **3** | **4** | **5** |
| Many unnecessary moves |  | Efficient time/motion but some unnecessary moves |  | Clear economy of movement and maximum efficiency |
| **Instrument Handling:** | | | | |
| **1** | **2** | **3** | **4** | **5** |
| Repeatedly makes tentative or awkward moves with instruments by inappropriate use of instruments |  | Competent use of instrument. but occasionally appeared stiff or awkward |  | Fluid moves with instruments and no awkwardness |
| **Knowledge of Instrument:** | | | | |
| **1** | **2** | **3** | **4** | **5** |
| Frequently asked for wrong instrument or used inappropriate instrument |  | Knew names of most instruments and used appropriate instrument |  | Obviously familiar with the instruments and their names |
| **Flow of Operation :** | | | | |
| **1** | **2** | **3** | **4** | **5** |
| Frequently stopped operating and seemed unsure of next move |  | Demonstrated some forward planning with reasonable progression of procedure |  | Obviously planned course of operation with effortless flow from one move to the next |
| **Use of Assistants:** | | | | |
| **1** | **2** | **3** | **4** | **5** |
| Consistently placed assistants poorly or failed to use assistants |  | Appropriate use of assistants most of the time |  | Strategically used assistants to the best advantage at all |
| **Knowledge of Specific Procedure:** | | | | |
| **1** | **2** | **3** | **4** | **5** |
| Deficient knowledge.  Needed specific instruction at most steps |  | Knew all important steps of operation |  | Demonstrated familiarity with all aspects of operation |
|  |  |  |  |  |

**OVERALL ON THIS TASK, SHOULD THE CANDIDATE: FAIL PASS**
